# Effects of Salt Stress on Three Ecologically Distinct *Plantago* Species

**DOI:** 10.1371/journal.pone.0160236

**Published:** 2016-08-04

**Authors:** Mohamad Al Hassan, Andrea Pacurar, María P. López-Gresa, María P. Donat-Torres, Josep V. Llinares, Monica Boscaiu, Oscar Vicente

**Affiliations:** 1 Institute of Plant Molecular and Cellular Biology (IBMCP, UPV-CSIC), Universitat Politècnica de València, Camino de Vera s/n, 46022, Valencia, Spain; 2 Research Institute for Integrated Coastal Zone Management (IGIC, UPV), Universitat Politècnica de València, C/ Paranimf 1, 46730, Gandia, Spain; 3 Mediterranean Agroforestal Institute (IAM, UPV), Universitat Politècnica de València, Spain, Camino de Vera s/n, 46022, Valencia, Spain; Institute of Genetics and Developmental Biology, Chinese Academy of Sciences, CHINA

## Abstract

Comparative studies on the responses to salt stress of taxonomically related taxa should help to elucidate relevant mechanisms of stress tolerance in plants. We have applied this strategy to three *Plantago* species adapted to different natural habitats, *P*. *crassifolia* and *P*. *coronopus*–both halophytes–and *P*. *major*, considered as salt-sensitive since it is never found in natural saline habitats. Growth inhibition measurements in controlled salt treatments indicated, however, that *P*. *major* is quite resistant to salt stress, although less than its halophytic congeners. The contents of monovalent ions and specific osmolytes were determined in plant leaves after four-week salt treatments. Salt-treated plants of the three taxa accumulated Na^+^ and Cl^-^ in response to increasing external NaCl concentrations, to a lesser extent in *P*. *major* than in the halophytes; the latter species also showed higher ion contents in the non-stressed plants. In the halophytes, K^+^ concentration decreased at moderate salinity levels, to increase again under high salt conditions, whereas in *P*. *major* K^+^ contents were reduced only above 400 mM NaCl. Sorbitol contents augmented in all plants, roughly in parallel with increasing salinity, but the relative increments and the absolute values reached did not differ much in the three taxa. On the contrary, a strong (relative) accumulation of proline in response to high salt concentrations (600–800 mM NaCl) was observed in the halophytes, but not in *P*. *major*. These results indicate that the responses to salt stress triggered specifically in the halophytes, and therefore the most relevant for tolerance in the genus *Plantago* are: a higher efficiency in the transport of toxic ions to the leaves, the capacity to use inorganic ions as osmotica, even under low salinity conditions, and the activation, in response to very high salt concentrations, of proline accumulation and K^+^ transport to the leaves of the plants.

## Introduction

Drought and soil salinity belong to the environmental factors most adverse for plants, which cause the biggest losses in agricultural production throughout the world and determine to a large extent the distribution of wild species in nature [1, 2]. Although the vast majority of plants, including all major crops, are glycophytes (that is, salt sensitive), there are also some species naturally adapted to saline environments, named halophytes. They represent only about 0.25% of total angiosperm taxa, but are very diverse from a taxonomic point of view, as tolerance to salinity seems to have evolved independently in different plant lineages [3].

Stress tolerance in plants relies on the activation of a series of conserved response pathways, some of them common to different abiotic stresses. One of these basic response mechanisms is the control of ion homeostasis and maintenance of osmotic balance, to counteract cellular dehydration caused by soil salinity, drought, cold or high temperatures, among other stressful conditions. The synthesis and accumulation of compatible solutes ('osmolytes') in the cytoplasm, in general, together with compartmentalisation of toxic ions in the vacuole when referring specifically to salt stress, are essential for osmotic adjustment [4–7]. Yet, the activation of these processes is not specific for halophytes, but shared by all plants, and does not necessarily result in salt tolerance [7]; in fact, as mentioned above, most species are sensitive to salinity. Therefore, the relative resistance to salt stress of plants, which varies widely among taxa, should be attributed to quantitative rather than qualitative differences in their mechanisms of response, which only in halophytes are efficient enough to confer salt tolerance, always within species-specific limits [7–10].

Several authors have proposed that halophytes possess constitutive mechanisms which enable their 'stress-anticipatory preparedness' or a metabolic anticipation of stress [11–12], a hypothesis supported by several experimental results. In a Mediterranean salt marsh in SE Spain no significant seasonal changes were detected in the levels of Na^+^, Cl^-^ or the osmolyte glycine betaine in two highly tolerant succulent halophytes, *Sarcocornia fruticosa* and *Inula crithmoides*, despite strong oscillations in soil water content and salinity during the two-year field study [13]. Moreover, the profiles of some major organic solutes in *Limonium latifolium* (a halophyte of the Plumbaginaceae family) upon NaCl treatments suggested that they pre-accumulate in the plants in prevention to stress [14]. Similarly, metabolite profiling indicated several-fold higher pre-stress concentrations of the major compounds related to salt tolerance in the halophyte *Thellungiella halophila* than in *Arabidopsis thaliana* [11]; the two species are somewhat related genetically, as both genera belong to the same family, Brassicaceae. Yet, the pre-adaptation hypothesis is still under debate; for example, comparative ionomic and metabolomic studies in three species of genus *Lotus* (Fabaceae), one of which is a moderate halophyte and two are typical glycophytes, did not reveal any 'preparedness to stress' in the halophyte [15]. For this reason, further studies on taxonomically related, stress tolerant and stress sensitive taxa–such as congener species; the closer the relation, the better–are of great interest and should be extended and diversified. In addition, these comparative studies would help to establish the relative contribution of different stress responses to the stress tolerance of a given species, by correlating the relative degree of stress resistance with the levels of biochemical markers associated to distinct stress response pathways–specific osmolytes, for instance [16]. One of the genera most appropriate for such comparative studies is probably *Plantago* L., with more than 200 species, including about 20 halophytes. The genus is well represented in the Mediterranean region, and 27 species, including several halophytes, are present in the Iberian Peninsula alone [17].

Three *Plantago* species, presumably with different degrees of stress resistance, were chosen for the present study. Two of them are considered salt tolerant, but have different ecological requirements and occupy different habitats. *P*. *crassifolia* grows in coastal wetlands, on saline soils in the Mediterranean region. *P*. *coronopus* L. has a broader distribution, reaching the Atlantic region of Europe and west Asia, where it grows in habitats with a variable degree of salinity, and often on degraded soils; its presence is considered as an indicator of salinisation of marginal lands. This study also included *P*. *major* L., widely distributed in Europe, Asia and north of Africa, and naturalised throughout the world.

Regarding their characteristic habitats, *P*. *crassifolia* appears exclusively on moderately saline soils–in the range 100–300 mM NaCl, approximately–occupying interdunar depressions and borders of salt marshes. Outwards from salt marshes, its presence is decreasing and it is not found beyond saline areas. On permeable and more or less flooded soils, *P*. *crassifolia* is often associated with monocots, shaping the characteristic plant community *Schoeno nigricantis-Plantaginetum crassifoliae* Br.-Bl. in Br.-Bl., Roussine & Nègre 1952, that often shelters interesting taxa, such as endemic species of *Limonium*, or endangered orchids [18–19]. Due to its botanical and ecological interest, this community type was included in the Red Natura 2000, constituting a subhabitat of Mediterranean salt meadows (*Juncetalia maritimi*) [18–20]. *P*. *coronopus* is also a halophytic species e.g. [21–23], but has a high adaptive potential, supporting different environmental conditions of climate, soil and vegetation cover [24], which allows its presence in many habitats and in association with a large number of species. In the Mediterranean coastal areas, it usually colonises degraded and compacted soils, rich in organic matter. *P*. *coronopus* also grows on soils with low or moderate salinity, subjected to trampling at salt marshes edges, where it may come in contact with *P*. *crassifolia*. However, *P*. *coronopus* also forms communities on non-saline, disturbed lands. It grows as well on the margins of freshwater wetlands, but with increasing water logging this species recedes in favour of the more hydrophilic *Plantago major*. The latter taxon usually appears in grasslands poor in species, on wet nitrified soils, with little exposure to sunlight [19]. Although a few *P*. *major* subspecies are adapted to saline environments [25], the common taxon *P*. *majo*r subsp. *major* included in the present study is frequent in humid areas, such as meadows and lawns, but also road edges. Since it is never found in salty soils, it is generally considered as a glycophyte, as reported for example in a detailed study on the ecology of the genus *Plantago*, carried out in a large number of natural habitats in the Netherlands [26]. Molecular taxonomy of *Plantago*, based on nuclear ribosomal ITS and plastid *trnL-F* sequences, recognises several clades with category of subgenera [27]. *P*. *crassifolia* and *P*. *coronopus* are closely related, belonging to subgenus *Coronopus* (Lam. & DC.) Rahn, whereas *P*. *major* is included in subgenus *Plantago*.

Different aspects of the responses to salinity have been previously investigated in a few *Plantago* species, especially in the halophyte *P*. *maritima* [28], often in comparison with non-halophytes from the same genus [29–33]. *P*. *crassifolia* has already been one of the target species of our studies [13, 34–38], but we are not aware of any comparative study on the salt tolerance of these three species in relation to their ecology and distribution in natural habitats.

In this paper we describe the effects of salt on the aforementioned *Plantago* species under controlled experimental conditions, regarding growth inhibition and the activation of basic stress responses based on the control of ion homeostasis and osmolyte accumulation. 'Shock' treatments with very high NaCl concentrations, beyond those the plants will normally encounter in the field, were included in the study to detect potential mechanisms which could be activated in response to an increase in the degree of environmental stress affecting the plants in their natural habitats–due, for example, to the foreseeable effects of climate change in the Mediterranean region. The specific aim of the work was to investigate the mechanisms underlying the tolerance to salinity in *Plantago*, by correlating the relative sensitivity to salt stress of the investigated species with the levels of ions and specific osmolytes accumulated in the leaves of the plants upon the salt treatments. A question of special interest that we also addressed was whether the relative tolerance to salinity of the three species, estimated from growth inhibition measurements under controlled conditions, corresponded to their distribution in different natural habitats.

## Material and Methods

### Plant material and experimental design

Seeds of *P*. *crassifolia* and *P*. *coronopus*, harvested from the Natural Park 'La Albufera' (Province of Valencia, Spain), were obtained from the seed stocks of the ‘Servicio Devesa-Albufera’, the department responsible for management of the natural park, and those of *P*. *major* were purchased from a commercial supplier (Spicegarden, EU); all seeds were stored at room temperature until used. Previous to germination, seeds were sterilised in a 5% hypochlorite solution (commercial bleach) for five minutes, and then washed thoroughly with distilled water. Germination was carried out on standard Petri dishes at 25°C and 16 hours photoperiod. After three weeks, seedlings were individually transplanted onto a moistened mixture of peat (50%), perlite (25%) and vermiculite (25%) in 1 L pots (Ø = 11 cm), placed in 55 x 40 cm plastic trays (12 pots per tray). During the entire course of plant growth the substrate was kept moist, by adding 1.5 L of Hoagland’s nutritive solution to each tray, twice a week. The salt treatments started three weeks after seedling transplantation, selecting ten seedlings of homogeneous size per species and per treatment; five pots of each set were treated for four weeks and the remaining five for eight weeks. Plants were watered twice a week with freshly prepared aqueous NaCl solutions of increasing concentration (100, 200, 400, 600, and 800 mM), using 1.5 L per tray. The control, non-treated plants were watered in parallel with distilled water. All experiments were conducted in a controlled environment chamber in the greenhouse, under the following conditions: long-day photoperiod (16 hours of light), temperature fixed at 23°C during the day and 17°C at night, and CO_2_ level of ≈300 ppm. Relative humidity ranged between 50 and 80% during the course of the treatments. A complete analysis of the responses of the plants to salt stress was carried out only with plant material harvested after one month treatment, since all plants died when maintained for two months in the presence or 600 or 800 mM NaCl.

### Soil analysis

Electrical conductivity (EC_1:5_) of the substrate was measured after four weeks of treatment. Soil samples were taken from pots of the same treatment, air-dried and then passed through a 2-mm sieve. A soil: water (1:5) suspension was prepared using deionised water at 20°C and mixed for one hour at 600 u/min. Electric conductivity was determined using a Crison Conductivity meter 522 and expressed in dS m^-1^.

#### Plant growth parameters

At the end of the treatments, the aerial part of each plant–constituted only by its rosette leaves, since during the course of the experiment the plants remained in the vegetative growth phase, before development of the reproductive stem–was collected and the following growth parameters were measured: number of leaves (NL), fresh weight (FW), and water content percentage (WC%). NL was recorded by simply counting the number of leaves of the plants under study. FW was measured by weighing the total mass of the leaves after harvesting. A fraction of the fresh material was dried in an oven at 65°C until constant weight, to obtain the dry weight (DW), which was used to calculate the leaf water content, in percentage, for each plant: WC = ((FW–DW)/ FW) x 100. Fresh plant material was stored frozen at -75°C, and dry material in tightly closed containers at room temperature. The collected plant samples included five replicates (five individual plants) per species and per treatment (control and different salt concentrations). All plants survived the four-week salt treatments, but the eight-week samples did not include plants treated with 600 or 800 mM NaCl, which did not survive the high-salt treatments.

#### Ion content measurements

Concentrations of potassium, sodium and chloride were measured in leaves of plants sampled after the stress treatments, and in the corresponding non-stressed controls. Extraction of K^+^ and Na^+^ were performed according to Weimberg [39], by heating the samples (0.15 g of dried, ground plant material in 25 mL of water) in a water bath, for 1 h at 95°C, followed by filtration through a filter paper (particle retention 8–12 μm); these cations were quantified with a PFP7 flame photometer (Jenway Inc., Burlington, USA). Chlorides were determined with the Spectroquant chloride test (Merck, Darmstadt, Germany).

### Osmolyte quantification

Proline (Pro) content was determined in fresh tissue by the ninhydrin-acetic acid method of Bates et al. [40]. Free Pro was extracted in 3% aqueous sulfosalicylic acid, the extract was mixed with acid ninhydrin solution, incubated at 95°C for 1 h, cooled on ice and then extracted with two volumes of toluene. The absorbance of the organic phase was determined at 520 nm using toluene as a blank. Glycine betaine (GB) was determined according to Grieve and Grattan [41] using dried tissue. Plant material was ground in a mortar, suspended in 2 ml of Milli-Q water and extracted with 1.2-dichlorethane; the absorbance of the solution was measured at a wavelength of 365 nm. Total sugars (TSS) were quantified according to the technique described by Dubois et al. [42]. Dried material was ground and mixed with 3 ml of 80% methanol on a rocker shaker for 24–48 h. Concentrated sulphuric acid and 5% phenol was added to the sample and the absorbance was measured at 490 nm. GB and TSS contents were only determined in leaf material of plants subjected to the four-week treatments, while Pro was also measured in the available eight-week samples.

Sorbitol (Sor) was analyzed using a Waters 1525 HPLC system coupled to a 2424 evaporative light scattering detector (ELSD). The source parameters of ELSD were the following: gain 75, data rate 1 point per second, nebulizer heating 60%, drift tube 50°C, and gas pressure 2.8 Kg/cm^2^. Plant dry material, from the four-week treatments, was boiled in milliQ water for 10 minutes and then filtered using 0.22 micrometer filters. Analysis was carried out injecting 20 μL aliquots with a Waters 717 autosampler into a Prontosil 120-3-amino column (4.6 x 125 mm; 3 μm particle size) maintained at room temperature. An isocratic flux (1 mL/min) of 85% acetonitrile during 25 minutes was applied in each run. Sor was identified and quantified by peak integration using the Waters Empower software and comparison with the standard calibration curve.

### Statistical analysis

Data were analyzed using the program Statgraphics Centurion v.16. Before the analysis of variance, the Shapiro-Wilk test was used to check for validity of normality assumption and Levene´s test for the homogeneity of variance. If ANOVA requirements were accomplished, the significance of the differences among treatments was tested by one-way ANOVA at a 95% confidence level and *post hoc* comparisons were made using the Tukey HSD test. All measured parameters in plants submitted to stress for four weeks, were correlated by principal component analysis (PCA), for each of the three studied *Plantago* species, using the program Statgraphics Centurion v.16. All means throughout the text are followed by SD.

## Results

### Electric conductivity of the soil

Soil electrical conductivity (EC_1:5_) values at the end of each stress treatment were similar for the three studied *Plantago* species, and in all of them salinity in the pot soil increased in parallel to the NaCl concentrations applied, as should be expected (Table 1). The only significant difference between species is the slightly higher EC measured in the pots of *P*. *major* plants growth in the absence of added salt, as compared with the control plants of the halophytes *P*. *crassifolia* and *P*. *coronopus* (Table 1).

**Table 1:** Increase in soil electric conductivity upon salt treatment. EC (dS m^-1^) in 1:5 water extracts (EC_1:5_) of pot soil samples after salt treatment of the plants. Values shown are means ± standard deviations (n = 5); different lower case letters in each column indicate statistically significant differences among treatments for the same species, and different capital letters in each row significant differences among species for each treatment, according to Tukey test (α = 0.05).

Treatment (NaCl)	*P*. *crassifolia*	*P*. *coronopus*	*P*. *major*
**0 mM**	**0.51 ± 0.04aA**	**0.50 ± 0.04aA**	**0.61 ± 0.04aB**
**100 mM**	**2.21 ± 0.13bA**	**1.97 ± 0.12bA**	**2.16 ± 0.11bA**
**200 mM**	**3.97 ± 0.28cA**	**4.11 ± 0.28cA**	**4.06 ± 0.20cA**
**400 mM**	**7.41 ± 0.38dA**	**6.90 ± 0.55dA**	**7.02 ± 0.56dA**
**600 mM**	**8.45 ± 0.27eA**	**8.47 ± 0.39eA**	**8.59 ± 0.36eA**
**800 mM**	**9.56 ± 0.66fA**	**9.44 ± 0.39fA**	**10.12 ± 0.72fA**

### Effect of salt stress on plant growth

**Fig 1:**
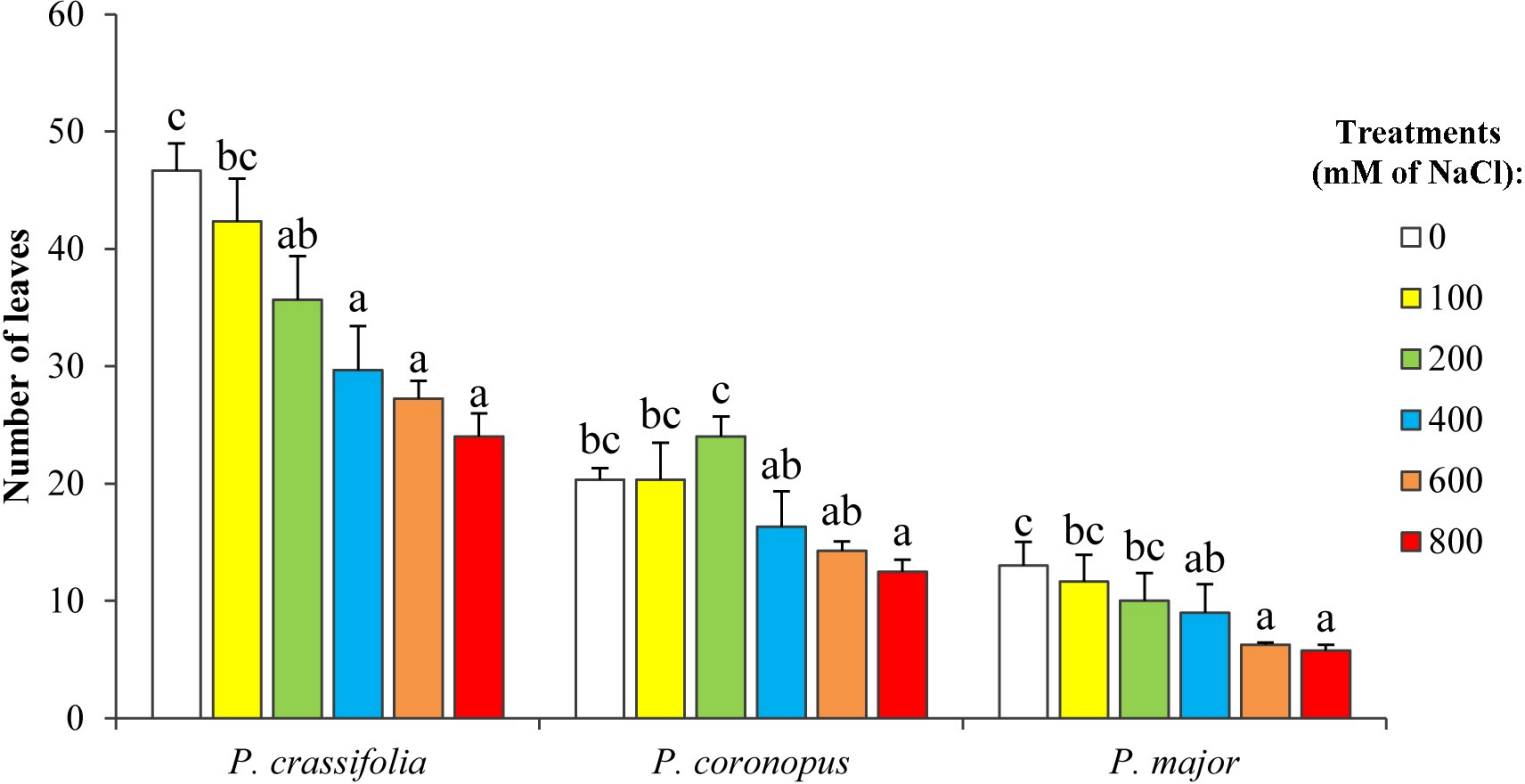
Salt-induced inhibition of plant growth. Number of leaves of *Plantago* plants after four weeks of treatement with the indicated NaCl concentrations (means ± SD, n = 5). Different lower case letters within each species indicate significant differences among treatments according to Tukey test (α = 0.05).

The most general, and the easiest to assess effect of stress on plants is inhibition of growth, which can be quantified by different measurements. A reduction in the number of leaves with increasing salt concentrations was detected in all selected *Plantago* species. The average number of leaves per plant varied from 12 (in *P*. *major*) to 45 (in *P*. *crassifolia*) for control, non-stressed plants, while at the highest salt concentration tested (800 mM NaCl) this number was reduced by 43% in *P*. *coronopus*, by 51% in *P*. *crassifolia* and by 54% in *P*. *major* (Fig 1). A similar pattern of reduction in the number of leaves, in relation to the non-treated controls, was observed in the plants that survived the eight-week salt treatments, up to 400 mM NaCl (see S1A Fig). Therefore, according to this criterion, *P*. *coronopus* appears to be the most salt-tolerant of the three species, which is also supported by the fact that a significant decrease in the mean leaf number was only observed in this species at high salt concentrations (≥ 400 mM NaCl, Fig 1, S1A Fig).

**Fig 2:**
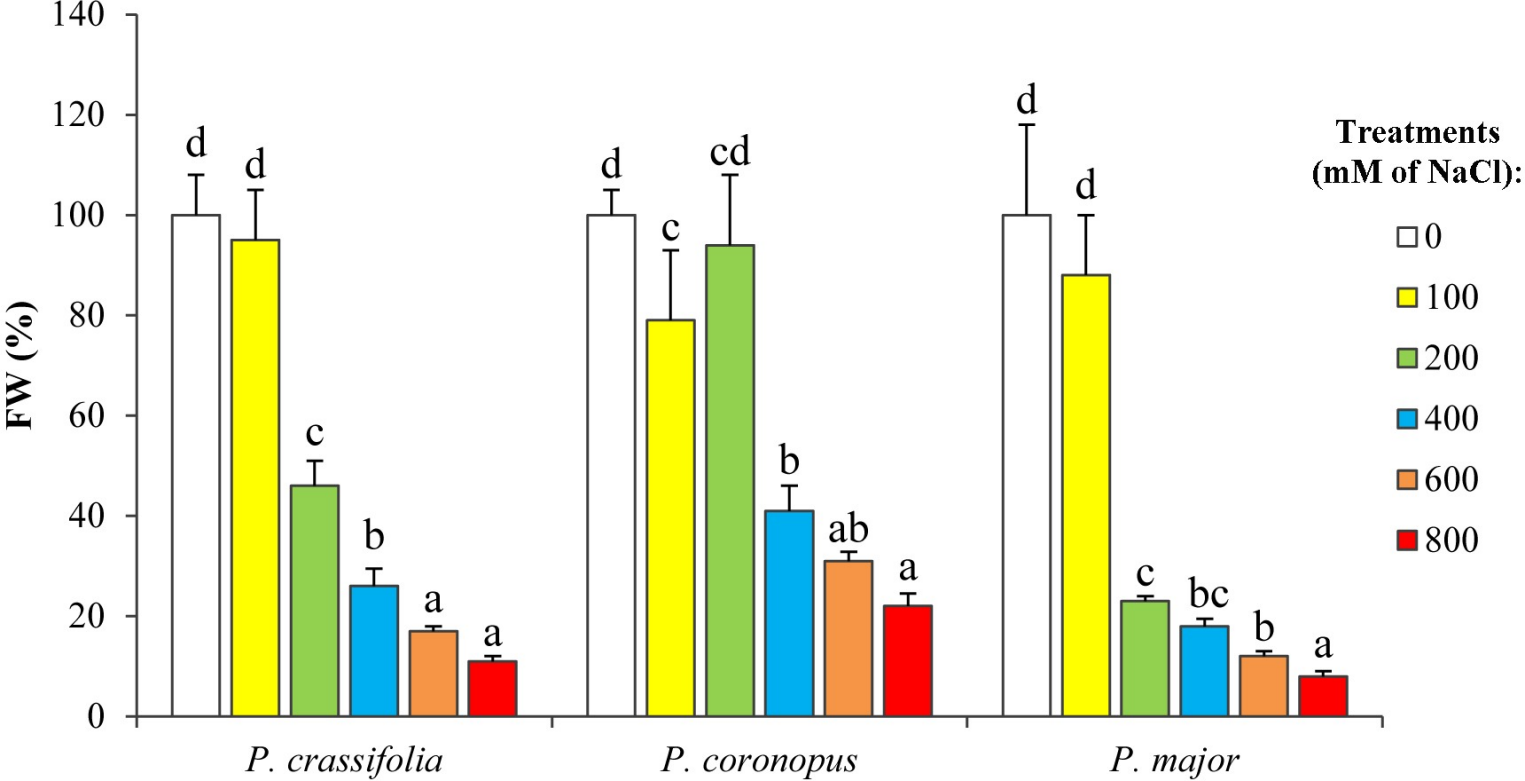
Inhibition of plant growth in the presence of increasing salt concentrations. Leaf fresh weight after four weeks of treatment with the indicated NaCl concentrations, for the three tested *Plantago* species. For each species, values (means ± SD, n = 5) are shown as percentages of the mean FW of the control plants, considered as 100% (absolute values: 11.35 ± 1.84 g, 10.61 ± 0.86 g, and 25.83 ± 3.51 g for *P*. *crassifolia*, *P*. *coronopus* and *P*. *major*, respectively). Different lower case letters within each species indicate significant differences among treatments according to Tukey test (α = 0.05).

The relative tolerance to salt of the three species was confirmed, and more clearly established by measuring the fresh weight of the leaves (after the four-week salt treatment), which also decreased in a concentration-dependent manner. In *P*. *crassifolia* and *P*. *major*, FW was significantly reduced already at 200 mM NaCl to ca. 45% and 20%, respectively, of the mean value of the corresponding controls; in *P*. *coronopus*, on the contrary, this salt concentration did not affect the fresh weight, and a significant decrease was only detected in the presence of 400 mM NaCl. Higher salt concentrations caused a stronger growth inhibition, but the relative sensitivity to NaCl of the three *Plantago* taxa was maintained (Fig 2), as it was after eight weeks of salt treatments (S1B Fig), where *P*. *major* plants showed again the largest reduction of fresh weight as compared to the corresponding non-stressed controls.

In the absence of salt, leaf water content–expressed as percentage of fresh weight (WC %)–varied from 85% in *P*. *major* to more than 95% in the succulent *P*. *crassifolia*, and decreased to some extent in the presence of salt for four weeks, as a result of increasing external NaCl concentrations, with a pattern similar for the three species (Table 2). Although the relative loss of water was slightly higher in *P*. *major* than in the other two species (19% *vs*. 13–14%), all three appear to possess efficient mechanisms to avoid dehydration of the leaves, even in the presence of very high salt concentrations. These data also indicate that the salt-dependent reduction of plant fresh weight in the three *Plantago* species was indeed due, mostly, to inhibition of growth, and that there were no important differences in their degree of water loss. The differences between the more salt-sensitive *P*. *major* and the halophytes became clearer when the salt treatments were prolonged: after eight weeks in the presence of 400 mM NaCl, *P*. *major* recorded a leaf water content of a mere 37%, while *P*. *crassifolia* and *P*. *coronopus* still showed very little dehydration (S1C Fig).

**Table 2:** Salt-induced leaf dehydration. Leaf water content (%) after four weeks of treatment with increasing NaCl concentrations, in the three selected *Plantago* species (means ± SD, n = 5). Different lower case letters within each species indicate significant differences among treatments according to Tukey test (α = 0.05).

Treatments (NaCl)	*P*. *crassifolia*	*P*. *coronopus*	*P*. *major*
**0 mM**	**95.41±0.20e**	**91.63±0.61e**	**84.54±2.20c**
**100 mM**	**94.33±0.24d**	**91.30±0.89be**	**82.50±1.09c**
**200 mM**	**92.13±0.39c**	**89.47±0.75d**	**81.61±1.13c**
**400 mM**	**86.39±3.76b**	**85.58±0.11c**	**72.70±0.91b**
**600 mM**	**85.89±1.23b**	**83.72±0.42b**	**71.35±1.11ab**
**800 mM**	**83.85±0.44a**	**78.45±0.59a**	**69.09±2.28a**

### Effects of salt stress on ion accumulation

**Fig 3:**
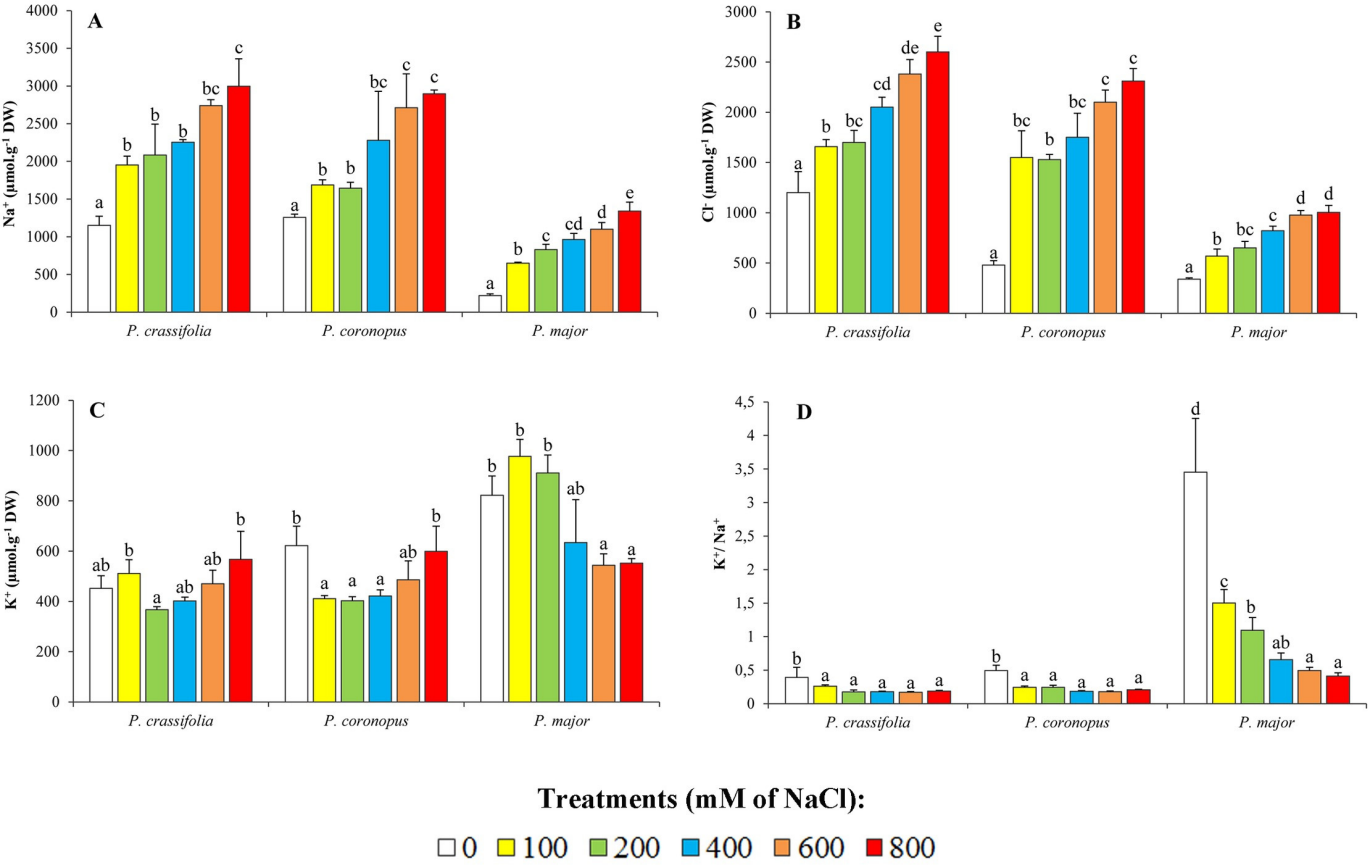
Monovalent ions in salt-treated plants. Leaf contents of (A) sodium (Na^+^), (B) chloride (Cl^-^), (C) potassium (K^+^), and (D) K^+^/Na^+^ ratios, in the selected *Plantago* species, after four weeks of treatment with the indicated NaCl concentrations (means ± SD, n = 5). Different lower case letters within each species indicate significant differences according to Tukey test (α = 0.05).

One common mechanism of salt tolerance in dicotyledonous halophytes is based on the transport of toxic Na^+^ and Cl^-^ ions to the leaves, where they accumulate in the vacuoles to avoid their deleterious effects in the cytosol, according to the so-called 'ion compartmentalisation hypothesis' [43–45]. Sodium levels in the leaves of NaCl-treated plants showed a concentration-dependent increase in the three species, although the absolute value reached in *P*. *major* in the presence of the highest salt concentration tested, 800 mM NaCl, (ca. 1.3 mmol g^-1^ DW) was less than half of those measured in the more tolerant taxa (2.8–3.0 mmol g^-1^ DW) (Fig 3A). It should be mentioned, however, that Na^+^ contents in *P*. *crassifolia* and *P*. *coronopus* untreated controls were relatively high, over 1 mmol g^-1^ DW, as compared to ca. 0.25 mmol g^-1^ DW in *P*. *major*; as a consequence, when comparing leaf Na^+^ levels at 800 mM external NaCl with the corresponding controls, the *relative* increase was higher in *P*. *major* (ca. 5.3-fold) than in *P*. *crassifolia* (2.7-fold) or *P*. *coronopus* (2.1-fold) (Fig 3A). The patterns of Cl^-^ accumulation in leaves and the absolute values reached at the highest salt concentrations tested were similar to those of Na^+^, in the three species; the only remarkable difference was that only *P*. *crassifolia*–but not *P*. *coronopus*–showed high Cl^-^ contents in the control plants not subjected to salt stress (Fig 3B). Similar qualitative patterns of Na^+^ and Cl^-^ accumulation in the three *Plantago* species were observed in plants treated with up to 400 mM NaCl for eight weeks (S2A and S2B Fig).

An increase in Na^+^ contents is generally accompanied by a concomitant decrease in K^+^ levels, as both cations compete for the same transport systems [46–47]. This general trend was observed in the three *Plantago* species, but with clear differences depending on their relative stress tolerance. In *P*. *crassifolia* and *P*. *coronopus*, a reduction of the mean K^+^ levels was observed at low and moderate NaCl concentrations, to increase again under high salinity conditions; *P*. *major*, on the other hand, showed relatively higher K^+^ contents in the absence of salt or below 400 mM NaCl, and a significant decrease was only observed at higher salt levels. In any case, variation of K^+^ concentrations in the plants was relatively small, with all values included in the range 0.4–1.0 mmol g^-1^ DW (Fig 3C). The relative levels of K^+^ and Na^+^ in each species led to two clearly different patterns of variation in K^+^/Na^+^ ratios. In control *P*. *major* plants, K^+^/Na^+^ was found to be quite high (ca. 3.5) and this value was progressively reduced, in parallel with the increase of salt concentrations, to 0.4 –that is, almost 9-fold–in the presence of 800 mM NaCl. In *P*. *crassifolia* and *P*. *coronopus*, K^+^/Na^+^ ratios were maintained below 0.5, with a reduction to about 50% of the controls in all salt treatments (Fig 3D). Here again, similar results were obtained after eight weeks of salt treatments, although the relative increase in K^+^ levels in the salt-tolerant taxa at very high salt concentrations could not be confirmed, since the plants did not survive in the presence of 600 and 800 mM NaCl (S2C and S2D Fig).

### Salt stress-induced osmolyte accumulation

**Fig 4:**
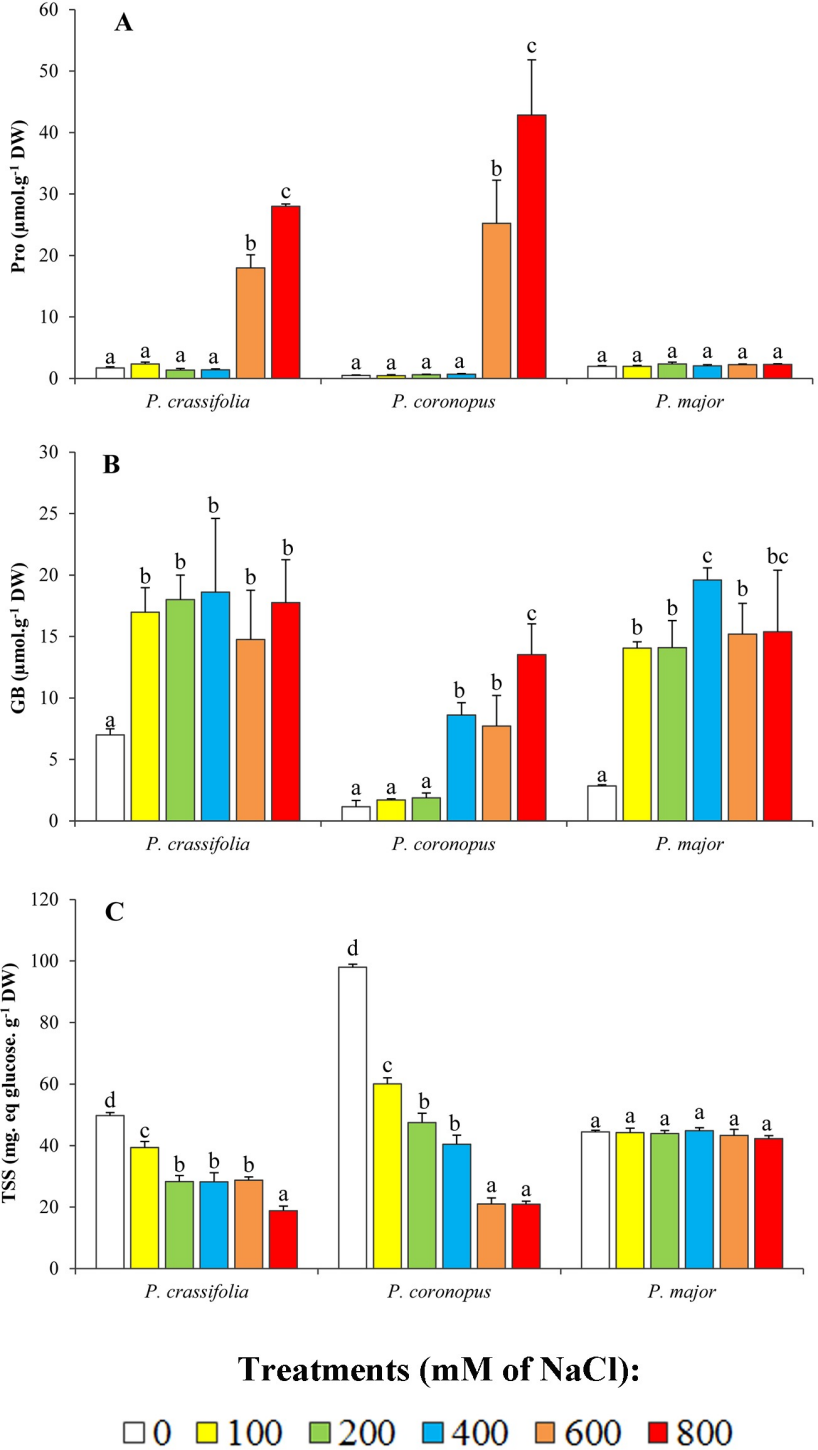
Osmolyte levels in salt-treated plants. Leaf contents of (A) proline (Pro), (B) glycine betaine (GB), and (C) total soluble sugars (TSS) in the selected *Plantago* species, after four weeks of treatment with the indicated NaCl concentrations (means ± SD, n = 5). Different lower case letters within each species indicate significant differences according to Tukey test (α = 0.05).

The levels of the commonest osmolytes in plants–proline (Pro), glycine betaine (GB) and total soluble sugars (TSS)–were determined in plant leaves after the salt treatments were concluded. Pro contents showed no significant increase over the non-stressed controls in the presence of NaCl up to 400 mM, in all three *Plantago* species. Yet stronger salt stress conditions triggered the accumulation of this osmolyte in *P*. *crassifolia*, at levels ca. 16-fold higher than in the control plants, in the presence of 800 mM NaCl; the induction of Pro biosynthesis was even stronger in *P*. *coronopus* (90-fold), while no increase over the control was detected in *P*. *major* under the same conditions (Fig 4A). It should be mentioned that, despite these strong relative increases in Pro contents in the most salt-tolerant species, even the highest absolute values reached, which were all below 50 μmol g^-1^ DW, are too low to contribute significantly to osmotic balance in the salt-stressed plants. The same is true for GB, which accumulated in the leaves of *Plantago* plants to maximum concentrations of around 20 μmol g^-1^ DW (Fig 4B), although with differences in the control levels and the patterns of accumulation: in *P*. *crassifolia* and *P*. *major*, GB synthesis was induced already in the presence of 100 mM NaCl and the contents of the osmolyte did not increase significantly at higher salt concentrations; in *P*. *coronopus*, GB levels increased more progressively, but only at and above 400 mM NaCl (Fig 4B). Total soluble sugars levels decreased moderately in *P*. *coronopus* and *P*. *crassifolia* with increasing NaCl concentrations, while no significant change was detected in *P*. *major* (Fig 4C). It appears, therefore, that these compounds do not play any role as osmolytes in the mechanisms of salt tolerance in the investigated *Plantago* species.

**Fig 5:**
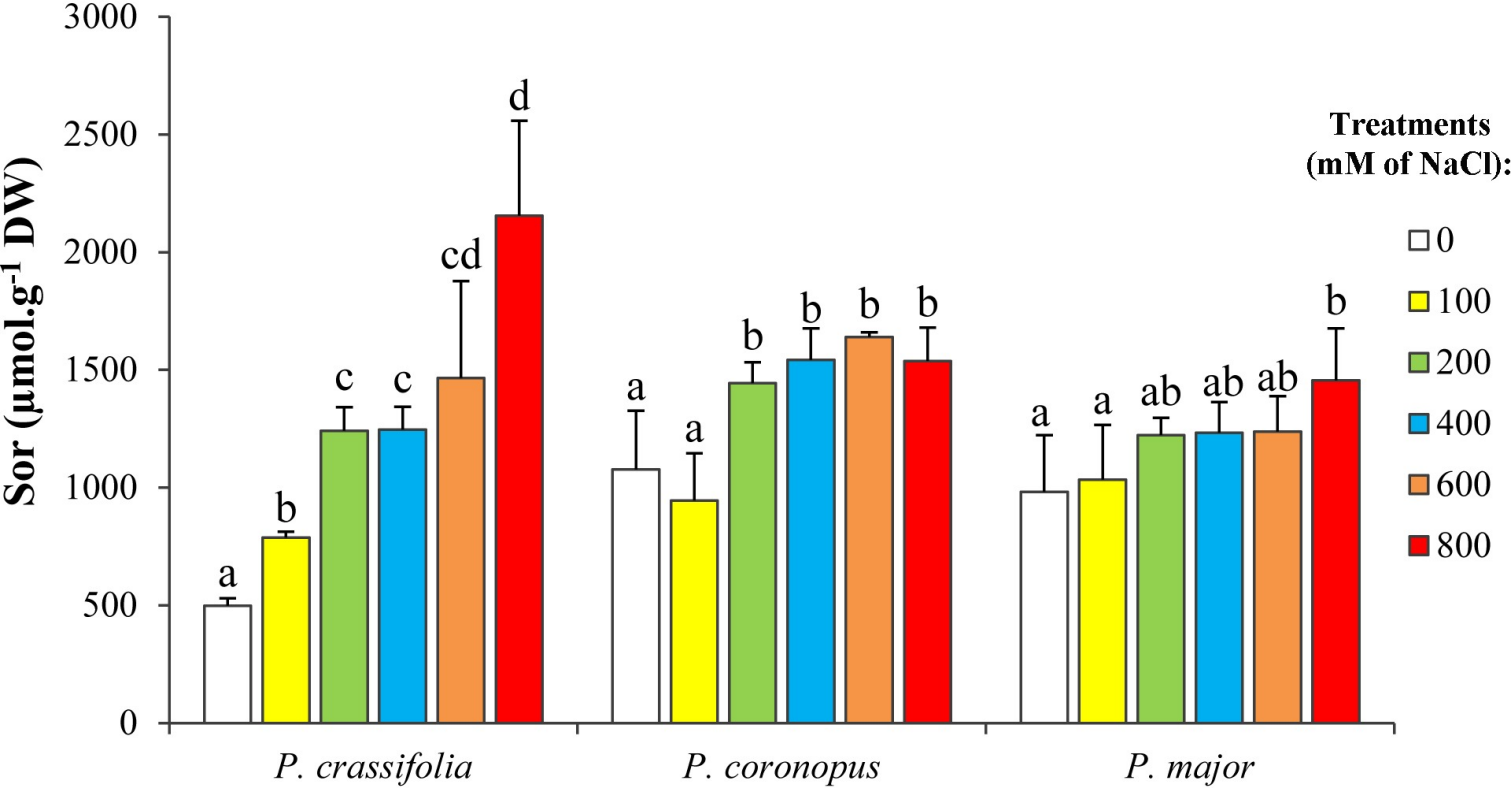
Sorbitol accumulation upon salt treatments of *Plantago* plants. Sorbitol (Sor) leaf contents after four weeks of treatement with the indicated NaCl concentrations, in the three selected *Plantago* species (means ± SD, n = 5). Different lower case letters indicate significant differences among treatments according to Tukey test (α = 0.05).

The spectrophotometric assay used to measure total soluble sugars does not detect polyalcohols [48] such as sorbitol (Sor), which has been identified as the major osmolyte in all *Plantago* species investigated to date [3, 30, 36, 49–51]. This has been confirmed in the present study, which shows that upon treatment with NaCl, Sor levels–identified and quantified by HPLC–gradually increased in leaves of all tested *Plantago* species, in a concentration-dependent manner (Fig 5), although some differences were observed among the three taxa. Sor contents in non-stressed *P*. *crassifolia* plants (ca. 500 μmol g^-1^ DW) were about half of those measured in *P*. *coronopus* and *P*. *major* controls, while the maximum level reached in the first species, in the presence of 800 mM NaCl, was higher than in the other two. Therefore, the relative salt-induced increase in the osmolyte concentration was highest in *P*. *crassifolia*: ca. 4.6-fold *vs*. 1.5-fold in *P*. *coronopus* and *P*. *major* (Fig 5). In any case, the absolute Sor concentrations were about three orders of magnitude higher than those of Pro or GB, supporting the essential role of Sor in cellular osmotic adjustment under salt stress conditions in *Plantago*.

### Principal Component Analyses

**Fig 6:**
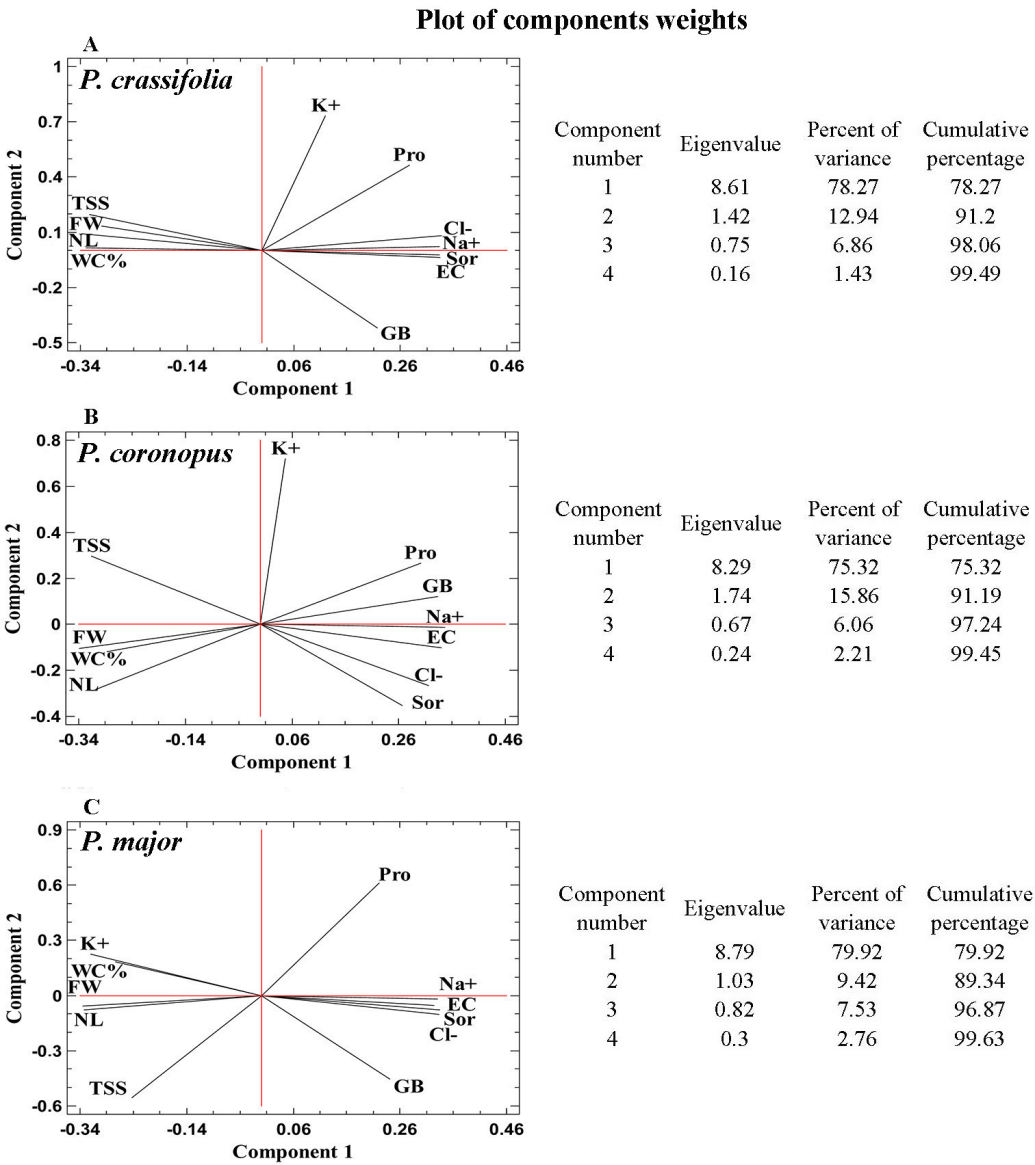
Principal Component Analyses (PCAs). Changes in all measured growth parameters and biochemical stress markers were analyzed in salt-treated plants with respect to the control, non-stressed plants of *Plantago crassifolia* (A), *P*. *coronopus* (B) and *P*. *major* (C), in correlation to EC (electrical conductivity) of the substrate, at the end of the four-week treatment. Abbreviations: number of leaves (NL), fresh weight percentage (FW%), water content percentage (WC%), sodium ions (Na^+^), potassium ions (K^+^), chloride ions (Cl^-^), sorbitol (Sor), proline (Pro), glycine-betaine (GB), total soluble sugars (TSS).

Principal component analyses (PCAs), including all measured parameters, were performed separately for plants of *P*. *crassifolia*, *P*. *coronopus* and *P*. *major*, sampled after one month of salt treatments. In the three PCAs shown in Fig 6, two components with an Eigenvalue equal to or greater than 1 explained a cumulative percentage of variance of about 90%. The first component (X-axis) was determined by the EC of the substrates (shown in Table 1), and alone explains 75–80% of the variance. Substrate salinity is strongly correlated, negatively, with the growth parameters analyzed (number of leaves, fresh weight and water content), and positively with toxic ions (Na^+^ and Cl^-^) and the major functional osmolyte, sorbitol, as shown by the small angles of the loading vectors of these variables with the X-axis. This is in agreement with the observed inhibition of growth and accumulation of ions and sorbitol in response to incresing NaCl external concentrations. A positive correlation, albeit weaker, was also registered between Pro and GB and the X-axis. The joint analysis of all variables revealed, in general, very similar patterns in the three studied taxa, indicating that they activate the same mechanisms in response to salt stress, and that differences between the halophytes and the more salt-sensitive species must be of a quantitative rather than a qualitative nature–accordingly with the ideas and assumptions mentioned in the introduction. The only clear difference between the three species is that K^+^ is strongly negatively correlated with EC of the substrate in *P*. *major* (K^+^ levels decrease with increasing salinity), but not in the two halophytes, where the variation of K^+^ is rather independent of the concentration of salt (K^+^ contents decrease al low and moderate salt concentrations, but increase at higher external salinity); this highlights the relevance of the activation of K^+^ transport to the leaves in the mechanisms of salt tolerance in *Plantago*.

## Discussion

### Effect of salt stress on plant growth

Growth of all glycophytes and most halophytes is optimal in the absence of salt and is progressively reduced by increasing salt concentrations; only in some highly tolerant dicotyledonous halophytes, growth is stimulated by low or moderate salinity, although still inhibited in the presence of salt above a higher, species-specific concentration threshold [52–54]. Therefore, a quantitative assessment of salt-induced growth inhibition in different species should provide a reliable ranking of their relative degree of salt tolerance. This kind of analysis, applied to the three *Plantago* species included in the present study, indicated that *P*. *major* is more sensitive to salt than *P*. *crassifolia* and *P*. *coronopus*, the latter apparently being the most tolerant; this would roughly agree with the distribution of the three taxa in nature: *P*. *crassifolia* and *P*. *coronopus* are both halophytes, although adapted to different types of saline habitats–and also non-saline, in the case of *P*. *coronopus*–while *P*. *major* is considered as a glycophyte, never found in natural saline environments. Yet, quantitative differences in growth parameters of the three species were relatively small, and *P*. *major* plants actually survived for four weeks the treatment with very high NaCl concentrations (600–800 mM), and for eight weeks in the presence of 400 mM NaCl. We have previously shown, in an independent study [34], that after two months exposure to NaCl up to 400 mM, *P*. *major* plants still produced some fertile flowers that generated viable seeds. Considering the accepted definition of halophytes as those plants able to complete their life cycle at soil salinity levels equivalent to at least 200 mM NaCl [55], *P*. *major* behaves as a moderate salt tolerant species, rather than as a typical glycophyte. Nevertheless, as it will be discussed below, the present work revealed clear differences in the responses to salt stress of this species and its two more resistant congeners.

Soil salinity is considered a major restrictive factor for plant survival in nature, so that only halophytes are adapted to highly saline soils, conditions that are lethal for non-tolerant species. However, our results indicate that the degree of stress tolerance, *per se*, is not the only factor–perhaps not even the most important factor–for the distribution of these species in their natural environments. Other components, such as interspecies competition, may play a fundamental role [56]. Many halophytes were probably refugees, outcompeted by glycophytes from non-saline areas, which remained highly competitive under saline conditions suboptimal for non-tolerant species. The three taxa analyzed here grew better in the absence of salt, but all tolerated relatively high NaCl concentrations. Yet it is likely that only *P*. *crassifolia* and *P*. *coronopus* are more competitive under saline conditions, whereas *P*. *major*, with a faster growth rate, may be more competitive in the absence of salt. This could explain why *P*. *crassifolia* and *P*. *coronopus*, although showing optimal growth in the greenhouse in the absence of salt, are found in nature in stressful environments, just the opposite to *P*. *major*, that never occurs in saline habitats, but has proven to be relatively salt tolerant.

### Effect of salt stress on ion accumulation

One basic response of glycophytes and monocotyledonous halophytes to cope with high salinity in the soil is the limitation of sodium uptake. On the contrary, dicotyledonous halophytes tend to accumulate toxic ions in the plants’ aerial parts, which are maintained at low cytsolic concentrations by compartmentalisation in vacuoles. This is an advantageous mechanism to increase osmotic pressure in foliar tissues, cheaper, in terms of energy consumption, than the synthesis of organic solutes for osmotic adjustment [57]. In many salt-tolerant plants, Na^+^ concentrations are often well above 200 mM on a tissue-water basis, concentrations that completely inhibit the activity of many enzymes *in vitro* [6]. In the present work, the highest levels of Na^+^ and Cl^-^ in leaves were found in *P*. *crassifolia*, followed by *P*. *coronopus*, and the lowest in the less tolerant *P*. *major*. It has been previously reported that non-halophytic *Plantago* species kept lower foliar Na^+^ levels under saline conditions than the halophyte *P*. *maritima* [30]. The ability of *P*. *maritima* to accumulate sodium in its shoots was based on its higher capacity of Na^+^ translocation from the roots, rather than on a higher uptake rate, which was similar to that found in other species of this genus [29, 58]. Differences in salt tolerance of *Plantago* species were also related to the efficiency of intracellular ion compartmentalisation mechanisms; for example, Staal *et al*. [33] reported a considerably greater tonoplast Na^+^/H^+^ antiporter activity under salt stress in the halophyte *P*. *maritima* than in the salt-sensitive *P*. *media*. The higher concentrations of Na^+^ and Cl^-^ we have determined in leaves of *P*. *crassifolia* and *P*. *coronopus*, as compared to *P*. *major*, are also related to the anatomic structure of the former species, with succulent leaves (especially in *P*. *crassifolia*), and therefore with increased vacuole volume and better ion sequestration capacity. Succulence, as well as excretion of Na^+^ and Cl^-^ by salt glands or bladders, is a basic anatomic adaptation to salinity in some dicotyledonous plants [52]. Yet, the most interesting information provided by these experiments is probably that the most tolerant taxa accumulated relatively high concentrations of Na^+^ (and also of Cl^-^, in the case of *P*. *crassifolia*) in the leaves of control, non-treated plants, whereas the more salt sensitive *P*. *major* did not. This could explain the statistically significant differences in electric conductivity of the substrate in the pots of the plants grown in the absence of salt, which was higher for *P*. *major* than for the two halophytes (Table 1). These data clearly suggest the existence in *Plantago* of constitutive mechanisms of tolerance, based on the active uptake and transport of sodium to the leaves, even under low salinity conditions, where the cation will be used as osmoticum–in addition to the accumulation, if necessary, of organic osmolytes–thus lending support to the halophytes’ 'pre-adaptation to stress' hypothesis [11–12]. The accumulation of sodium in plants is generally associated with a decrease of potassium levels in the shoots. Since the two cations compete for the same binding sites, Na^+^ interferes with K^+^ transport into the cell by using its physiological transport systems [9, 43, 52]. Moreover, sodium uptake produces a depolarization of the plasma membrane causing the activation of outward-rectifying K^+^ channels and consequently a K^+^ loss [59–60]. These processes lead to a significant reduction of K^+^/Na^+^ ratios, and maintenance of relatively high cellular K^+^/Na^+^ values appears to be a relevant mechanism of salt tolerance [61]. The three species showed a general trend of reduction of K^+^/Na^+^ ratios with increasing salinity, more accentuated in *P*. *major*. However the highest K^+^/Na^+^ value was found in the latter species, even under saline conditions, due to the higher Na^+^ accumulation in the leaves of the two halophytes–mostly in the vacuoles, presumably. The issue of how much Na^+^ accumulates in the cytosolic compartment under salt stress conditions is controversial and seldom discussed in the literature. Indeed, the value of cytosolic K^+^/Na^+^ ratio has been questioned, and a more parsimonious alternative view has been proposed, based just on the determination of K^+^ levels under salt stress [62]. Regarding K^+^ contents, there were clear differences between *P*. *crassifolia* and *P*. *coronopus*, on the one side, and *P*. *major*, on the other. It is especially interesting to note the significant increase of K^+^ levels observed in the more salt-resistant taxa at high external NaCl concentrations–under lower salinity conditions, a decrease in K^+^ was detected, as expected–suggesting the activation of potassium transport to the leaves, which could partly compensate the accumulation of sodium, thus contributing to salt tolerance. In the less tolerant *P*. *major*, K^+^ contents in the absence of salt were higher than in the other two taxa, and did not change significantly up to ca. 400 mM NaCl, which fits with the lower Na^+^ concentrations in the leaves; only at higher salinity levels a decrease of K^+^ levels was detected.

### Osmolyte synthesis

Environmental stress conditions leading to cellular dehydration, including salt stress, trigger the cytosolic accumulation of different organic compatible solutes, or osmolytes. The contribution of osmolytes to stress tolerance is not limited to their function in osmotic adjustment, as they have multiple additional roles as osmoprotectants, directly stabilising proteins and macromolecular structures under stress conditions, as ROS scavengers or, in some cases, as signalling molecules involved in the induction of changes in gene expression patterns [6, 55, 63–64]. In the genus *Plantago*, all available data indicate that the polyalcohol sorbitol is the major functional osmolyte [3, 50, 55, 65]. Sorbitol accumulation in response to salt treatments has been reported in the salt-tolerant *P*. *maritima* [29, 48–49] and *P*. *coronopus* [23, 65], as well as in the salt-sensitive *P*. *media* and *P*. *lanceolata* [30]. In one of our previous studies on *P*. *crassifolia*, we confirmed accumulation of this compound in plants growing in a littoral salt marsh in SE Spain, and showed how seasonal changes in sorbitol levels correlated with the degree of environmental stress affecting the plants in their natural habitat [36].

Sorbitol accumulated in response to salt stress, in the three species studied here. Yet, the relative increases over the corresponding controls were very poor, since sorbitol concentrations were already high in the absence of salt, especially in *P*. *coronopus* and *P*. *major* (about 1 mmol g^-1^ DW). Therefore, these species appear to accumulate the osmolyte in the absence of salt, so that a strong induction of its synthesis when the plants are actually affected by high salinity conditions is not necessary, and the regulation of the response to salt stress could be mostly based on changes in the intracellular localisation of sorbitol. This is obviously also in agreement with the idea of a ‘pre-adaptation’ or ‘metabolic preparedness’ to stress in tolerant species. In *P*. *crassifolia*, sorbitol content in the absence of salt was about 50% of that measured in *P*. *coronopus* or *P*. *major*–which could be compensated, in terms of osmotic balance, by the higher levels of monovalent ions, Na^+^ and Cl^–^–and the maximum absolute values measured at the highest NaCl concentration tested were somewhat higher; therefore, the relative level of salt-dependent accumulation of sorbitol was higher in this species. However, contrary to what was observed regarding accumulation of monovalent ions in non-stressed plants, sorbitol leaf levels in the absence of salt were relatively high in the three investigated species, irrespective of their tolerance. Therefore, although sorbitol must play a significant role in osmotic adjustment in all *Plantago* species, their relative degree of salt tolerance does not seem to be dependent on the differential accumulation of this osmolyte in the studied taxa.

Pro and GB are probably the commonest osmolytes in plants, used by many angiosperm species to help maintain cellular osmotic balance. In a study on 51 halophytes sampled in their natural environment in an inland salt marsh in Turkey, Tipirdamaz et al. [66] found these two osmolytes as almost omnipresent solutes. In *P*. *maritima* they detected even higher levels of Pro and GB than of sorbitol (which was also substantial), despite the fact, mentioned above, that sorbitol is considered the major functional osmolyte in the genus *Plantago*; this could be explained by biosynthesis of Pro and GB from O_2_-dependent metabolic pathways that would be promoted in non-submerged salt marshes, while sorbitol would accumulate under more anaerobic conditions, such as those present in marine salt marshes [66]. Pro accumulation in salt-treated *P*. *maritima* plants, in both roots and shoots, has also been recently reported [67]. The concomitant synthesis of different osmolytes has been previously observed in many halophytes [68], although it is generally assumed that each species uses preferentially one particular compound for osmotic balance under stress conditions. For example, typical GB accumulators first accumulate this compatible solute, and only later-on, when stress is more accentuated, they start to accumulate Pro [69]. A similar behaviour has been observed in the most salt-tolerant *Plantago* species included in the present study, *P*. *crassifolia* and *P*. *coronopus*, in which no significant Pro accumulation was observed at salinity levels equivalent to those present in their natural habitats, below 400 mM NaCl. Only at very high salt concentrations, 600–800 mM NaCl, large relative increases of Pro contents were detected. Pro accumulation is triggered specifically under high salinity conditions, but it is not induced by longer treatments at lower salt concentrations, as it was not observed in the plants maintained for two months in the presence of 400 mM NaCl (shown in S1 Table). In the more sensitive *P*. *major*, no induction of Pro biosynthesis was observed, at any of the tested salt concentrations. Some of these data confirm our previous Pro measurements performed in leaves from *P*. *crassifolia* plants grown in the field [13]. Accumulation of additional osmolytes under increased levels of salt stress, for example Pro in the *Plantago* salt tolerant taxa, may be a built-in mechanism which could enable halophytes to rapidly adapt to and withstand possible increases in the degree of salt stress in their natural habitats, either short-term temporary changes in soil salinity or long-term increased salinization, such as that that can be expected as a consequence of climate change in salt marshes of the Mediterranean basin and other arid and semiarid regions. It should be pointed out that the maximum Pro concentrations reached under the strongest stress conditions tested are still too low–three orders of magnitude below those of sorbitol–to contribute substantially to osmotic adjustment, even assuming that it accumulated exclusively in the relatively small volume of the cytoplasm. The same could be said of GB, which was found to slightly increase in the three investigated species, as a response to salt stress, but to reach maximum absolute contents below those of Pro, and much lower than those reported for plants that are true GB accumulators [13, 66, 70]. Nevertheless, it is likely that Pro, and maybe also GB, play a significant role in the tolerance mechanisms of these species in the presence of strong stress conditions. Yet these mechanisms would not involve maintenance of cellular osmotic balance, but rather be based on other functions as a low-molecular-weight chaperons, ROS scavengers and/or signalling molecules [10, 71–75]. Pro and GB, among other osmolytes, may also contribute to the reduction of K^+^ loss from the cell in the presence of high salt concentrations, since they have been reported to have additional roles in the protection of plasma membrane integrity and its associated transporter proteins [76–77].

## Conclusions

Distribution in nature of the *Plantago* taxa included in this study does not appear to depend only on their relative degree of salt tolerance, estimated from growth inhibition measurements, but largely on other factors–such as interspecific competition, for example. *P*. *major*, which is never found in natural saline environments, proved to be quite tolerant to salinity in our experiments, while the responses to salt stress of *P*. *crassifolia* and *P*. *coronopus* under controlled greenhouse conditions were almost identical, in qualitative and quantitative terms, even though these species are adapted to different types of saline habitats.

Our findings show that general responses to salt stress in *Plantago* mostly involve the transport of toxic ions to the leaves, their sequestration in the vacuole, and the presence of high sorbitol concentrations in the cytoplasm for osmotic balance. The relative tolerance of the three taxa is partly dependent on quantitative differences in the efficiency of these processes–for example, Na^+^ and Cl^-^ leaf contents are lowest in the most salt-sensitive *P*. *major*, indicating reduced ion transport from the roots. Yet, there also exist specific salt-stress responses characteristic of the more tolerant species–and therefore relevant for the mechanisms of salt tolerance–such as the capacity to use inorganic ions as osmotica, even under low salinity conditions. In addition, these salt-tolerant taxa seem to be better adapted to withstand strong increases in soil salinity: in the presence of very high NaCl concentrations, they accumulate relatively large amounts of a secondary osmolyte (Pro), and appear to activate K^+^ transport to the leaves, thus avoiding a drastic reduction in K^+^/Na^+^ ratios. Activation of these specific responses may be ecologically relevant, as they could represent built-in mechanisms allowing the plants to rapidly adapt to increasing salinity in their natural habitats, due for example to the effects of climate change in arid or semiarid regions.

Despite species-specific quantitative differences, some of these response mechanisms appear to be constitutive, as monovalent cations and sorbitol leaf contents are relatively high even in the absence of stress. Therefore, our results also lend support to the hypothesis of a ‘pre-adaptation’ to stress in tolerant species of the genus *Plantago*.

## Supporting Information

S1 Fig Salt-induced inhibition of plant growth. (A) number of leaves, (B) leaf fresh weight (%), (C) leaf water content (%) in the three analyzed *Plantago* species, after eight weeks of treatment with the indicated concentrations of NaCl (means ± SD, n = 5). FW values are shown as percentages of the mean FW of the control plants, considered as 100% (absolute weights: 66.25 ± 5.48 g, 29.52 ± 3.29 g, and 71.94 ± 7.78 g for *P*. *crassifolia*, *P*. *coronopus* and *P*. *major*, respectively). Different lower case letters within each species indicate significant differences between treatments, according to Tukey test (α = 0.05).
(TIF)

S2 Fig Monovalent ions in salt-treated plants. Leaf contents of (A) sodium (Na^+^), (B) chloride (Cl^-^), (C) potassium (K^+^), and (D) K^+^/Na^+^ ratios, in the selected *Plantago* species, after eight weeks of treatment with the indicated NaCl concentrations (means ± SD, n = 5). Different lower case letters within each species indicate significant differences between treatments, according to Tukey test (α = 0.05).
(TIF)

S1 Table Proline levels in salt-treated plants. Leaf contents of Pro (μmol g^-1^ DW), in the selected *Plantago* species, after eight weeks of treatment with the indicated NaCl concentrations (means ± SD, n = 5). Different lower case letters within each species indicate significant differences between treatments, according to Tukey test (α = 0.05).
(DOCX)
