# Supplementary figures and images for: Effects of Salt Stress on Three Ecologically Distinct Plantago Species
Source: PLoS One. 2016 Aug 4;11(8):e0160236. doi: 10.1371/journal.pone.0160236 (PMC4973956; doi:10.1371/journal.pone.0160236)

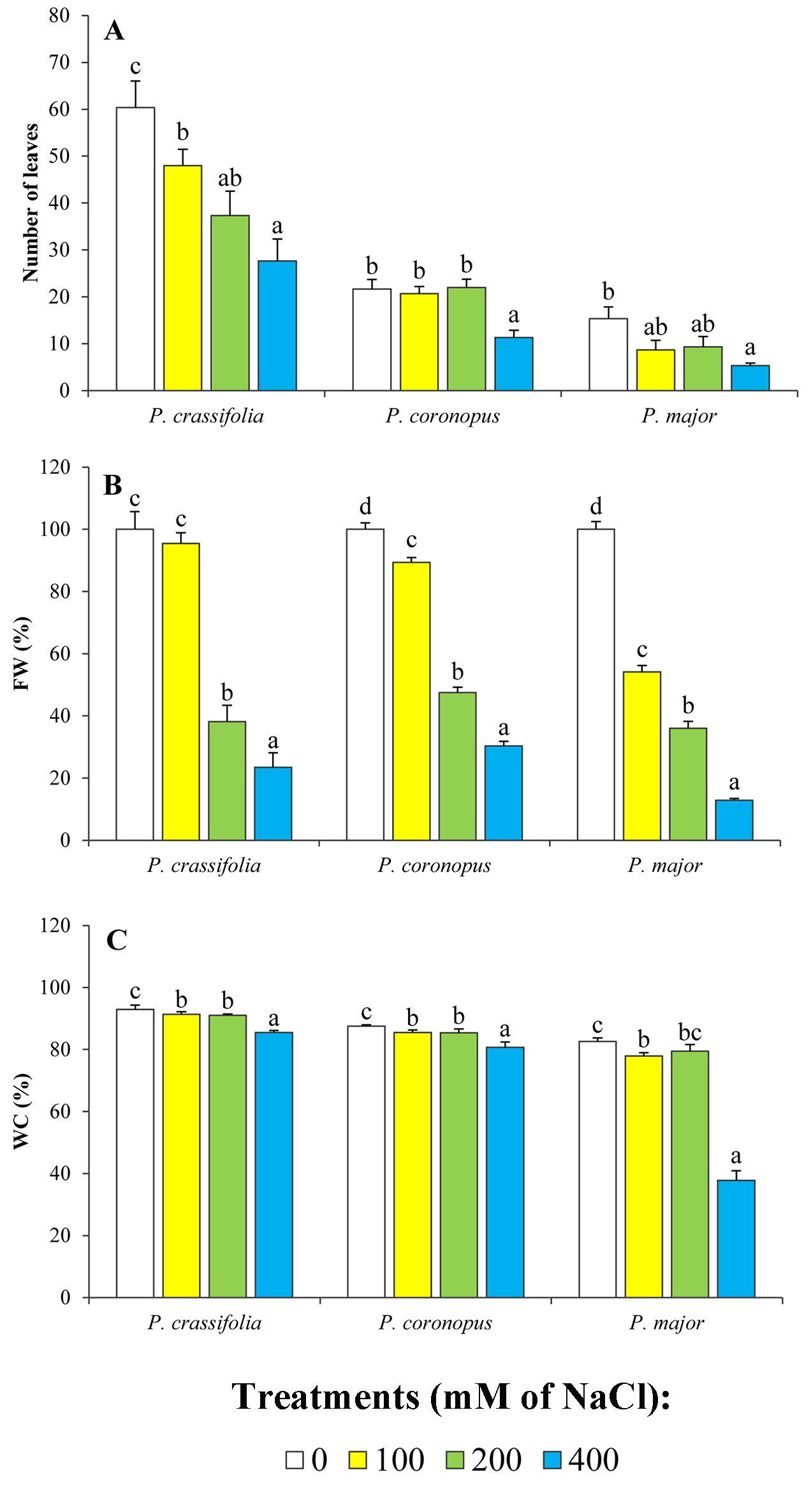

Supplement: S1 Fig — (A) number of leaves, (B) leaf fresh weight (%), (C) leaf water content (%) in the three analyzed Plantago species, after eight weeks of treatment with the indicated concentrations of NaCl (means ± SD, n = 5). FW values are shown as percentages of the mean FW of the control plants, considered as 100% (absolute weights: 66.25 ± 5.48 g, 29.52 ± 3.29 g, and 71.94 ± 7.78 g for P. crassifolia, P. coronopus and P. major, respectively). Different lower case letters within each species indicate significant differences between treatments, according to Tukey test (α = 0.05). (TIF) [file pone.0160236.s001.tif]

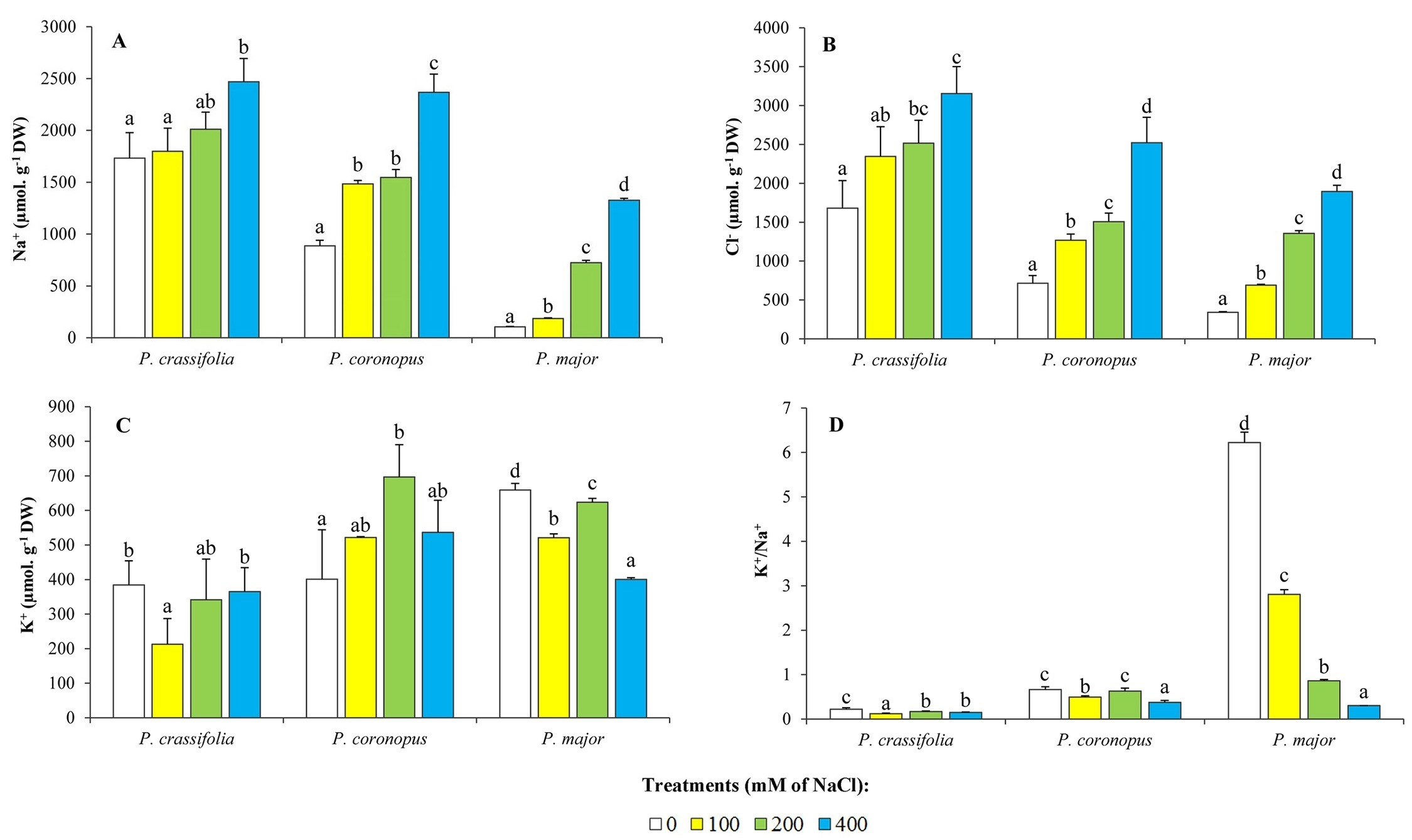

Supplement: S2 Fig — Leaf contents of (A) sodium (Na+), (B) chloride (Cl-), (C) potassium (K+), and (D) K+/Na+ ratios, in the selected Plantago species, after eight weeks of treatment with the indicated NaCl concentrations (means ± SD, n = 5). Different lower case letters within each species indicate significant differences between treatments, according to Tukey test (α = 0.05). (TIF) [file pone.0160236.s002.tif]
