# Supplementary material for: Effects of Salt Stress on Three Ecologically Distinct Plantago Species
Source: PLoS One. 2016 Aug 4;11(8):e0160236. doi: 10.1371/journal.pone.0160236 (PMC4973956; doi:10.1371/journal.pone.0160236)
Supplement: S1 Table — Leaf contents of Pro (μmol g-1 DW), in the selected Plantago species, after eight weeks of treatment with the indicated NaCl concentrations (means ± SD, n = 5). Different lower case letters within each species indicate significant differences between treatments, according to Tukey test (α = 0.05). (DOCX) [file pone.0160236.s003.docx]

**Table. S1 Proline levels in salt-treated plants.** Leaf contents of Pro (µmol. g^-1^ DW), in the selected *Plantago* species, after eight weeks of treatment with the indicated NaCl concentrations (means ± SD, n = 5). Different lower case letters within each species indicate significant differences between treatments, according to Tukey test (α = 0.05).

| Treatment (NaCl) | *P. crassifolia* | *P. coronopus* | *P. major* |
| --- | --- | --- | --- |
| 0 mM | 1.43±0.12a | 1.49±0.14a | 2.41±0.43bc |
| 100 mM | 1.73±0.15b | 2.33±0.27b | 2.11±0.21ab |
| 200 mM | 1.49±0.08a | 1.43±0.12a | 1.92±0.46a |
| 400 mM | 1.67±0.12ab | 1.44±0.21a | 3.19±0.43c |
